# Bibliometric Indicators of the Zika Outbreak

**DOI:** 10.1371/journal.pntd.0005132

**Published:** 2017-01-19

**Authors:** Priscila C. Albuquerque, Mauro Jorge C. Castro, Juliana Santos-Gandelman, Ana Claudia Oliveira, José M. Peralta, Marcio L. Rodrigues

**Affiliations:** 1 Fundação Oswaldo Cruz (Fiocruz), Centro de Desenvolvimento Tecnológico em Saúde (CDTS), Rio de Janeiro, Brazil; 2 Instituto de Microbiologia Paulo de Góes, Universidade Federal do Rio de Janeiro (UFRJ), Rio de Janeiro, Brazil; 3 Associação Brasileira das Indústrias de Química Fina, Biotecnologia e suas Especialidades–Abifina, Rio de Janeiro, Brazil; Aix Marseille University, Institute of Research for Development, and EHESP School of Public Health, FRANCE

## The Zika Outbreak

The current Zika outbreak and its obvious relevance to public health motivated important changes in the traditional process of peer review and publication of scientific articles. The public health emergency of international concern demanded rapidly available information, aiming to generate knowledge applicable for combating the crisis. Major scientific journals are now calling for papers on the Zika virus (Table 1), offering fast-track review of submissions that usually undergo a streamlined peer-review process followed by immediate publication upon acceptance of articles [1–5]. Scientific content concerning the Zika virus is usually free to access, which accelerates knowledge flow. In many journals, reviewers are asked to evaluate only if the research methods are sound and support the conclusions and if the work will contribute in some way towards resolving the immediate challenges [3]. This scenario induced a desirable upsurge in the generation of knowledge translated into scientific publications [6]. On the basis of our previous experience of mapping scientific trends in the field of fungal infections [7], bibliometric indicators of the Zika outbreak were analyzed, aiming to produce a general picture of where the field of Zika virus research currently stands.

**Table 1:** The accelerated flow of knowledge in the field of Zika virus: facilitated mechanisms for publication of scientific articles.

Publisher	Journal	Number of articles in the field (publication date of first article)	Mechanism of accelerated dissemination of knowledge and publisher website
American Association for the Advancement of Science	Science	30 (November 2015)	Research topic, http://www.sciencemag.org/topic/zika-virus
Elsevier	Acta Tropica	1 (April 2016)	Resource center, https://www.elsevier.com/connect/zika-virus-resource-center; http://www.cell.com/public-health-zika-virus; www.thelancet.com/campaigns/zika;
	American Journal of Obstetrics & Gynecology	1 (February 2016)	
	Antiviral Research	1 (June 2016)	
	Autoimmunity Reviews	2 (September 2016)*	
	Cell	8 (February 2016)	
	Cell Host & Microbe	16 (April 2016)	
	Cell Reports	7 (June 2016)	
	Cell Stem Cell	8 (March 2016)	
	Clinical Microbiology and Infection	1 (April 2016)	
	Current Opinion in Virology	1 (June 2016)	
	Diagnostic Microbiology and Infectious Disease	1 (September 2016)*	
	EBioMedicine	1 (September 2016)*	
	Emergency Medicine Clinics of North America	1 (August 2016)	
	Epidemics	1 (June 2016)	
	Infection, Disease & Health	1 (September 2016)*	
	Infection, Genetics and Evolution	1 (September 2016)	
	International Journal of Infectious Diseases	7 (February 2016)	
	Journal of Autoimmunity	1 (March 2016)	
	Journal of Clinical Virology	7 (July 2015)	
	Journal of Infection	2 (September 2016)*	
	Journal of Molecular Biology	1 (September 2016)*	
	Journal of Virological Methods	1 (October 2016)	
	Microbes and Infection	6 (December 2015)	
	New Microbes and New Infections	2 (February 2016)	
	The Lancet	29 (December 2015)	
	Transfusion Medicine Reviews	1 (September 2016)*	
	Travel Medicine and Infectious Disease	19 (July 2015)	
	Trends in Immunology	1 (September 2016)*	
	Trends in Microbiology	2 (May 2016)	
	Trends in Parasitology	1 (April 2016)	
	Vaccine	3 (April 2016)	
	Virology	3 (June 2016)	
NEJM Group	New England Journal of Medicine	22 (February 2016)	Collection, http://www.nejm.org/page/zika-virus
Oswaldo Cruz Foundation, Brazil	Memórias do Instituto Oswaldo Cruz	10 (June 2015)	Fast track, http://memorias.ioc.fiocruz.br/issues/zika-fast-track
Oxford University	Journal of Travel Medicine	1 (January 2016)	Collection
	Transactions of the Royal Society of Tropical Medicine & Hygiene	21 (September 1952)	Collection, http://www.oxfordjournals.org/en/our-journals/medicine-and-health/aedes-aegypti-zika-virus.html
	Clinical Infectious Diseases	7 (April 2016)	
	The Journal of Infectious Diseases	3 (May 2016)	
	Brain	1 (June 2016)	
Public Library of Science	PLOS Neglected and Tropical Diseases	34 (February 2012)	Collection, http://collections.plos.org/zika
	PLOS Biology	1 (July 2016)	
	PLOS Current Outbreaks	11 (June 2014)	
	PLOS ONE	2 (September 2014)	

* Articles that were in press at the time of the analysis (September 23, 2016).

## The Scientific Expansion of the Zika Virus Field

The last decades have seen outbreaks caused by a number of viruses, including Chikungunya, Ebola, and Dengue. Literature analysis indicates that these health emergencies were efficient catalyzers of the generation of scientific knowledge. For instance, only 8 articles were published in 2005 in the field of the Chikungunya virus [8]. After its confirmation as the cause of an epidemic of dengue-like illness on the Comoros Islands in 2005, the annual number of articles increased year by year to reach 302 in 2014 [8]. Ebola literature had an annual median number of articles of 43 before the West African outbreak in 2013. In 2014, more than 600 articles were published in the field [9]. The number of published documents in the field of Dengue climbed from less than 50 per year before the 1990s to almost 2,500 per year in 2015 [10].

**Fig 1:**
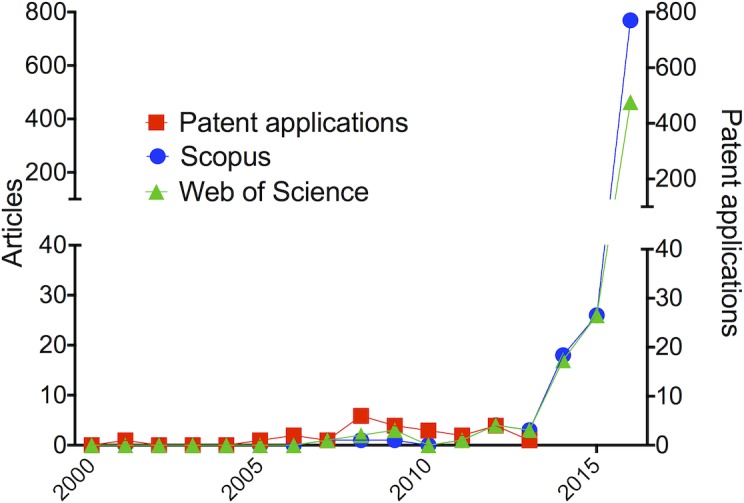
Patent application and publication records (January 1, 2000–August 31, 2016) containing the keyword “Zika virus” in article titles [14,15] or in patent application claims [18–21]. For analysis of raw data, see S1–S3 Tables.

From the initial isolation and serologic analysis of the Zika virus in Uganda in 1952 [11,12] to the outbreak in French Polynesia in 2013 [13], a few citable documents covering Zika infections were available. Simple searches in the Web of Science and Scopus literature databases [14,15] crossing the title words “Zika” and “French Polynesia” resulted in only 15 and 17 documents, respectively. From 1952 to 2013, articles containing the keyword “Zika virus” in their titles totaled 44 (Scopus) and 28 (Web of Science) documents. From January 2014 to August 2016, this number dramatically increased (Fig 1, S1 and S2 Tables, and [6,16]). Considering that Zika has historically been a neglected tropical disease, we also included in our analysis a general search to include non-peer-reviewed literature. Similar profiles were found using the Google Scholar search engine [17], which revealed 47 documents containing the title words “Zika virus” from 1952 to 2013 and approximately 1,600 documents between 2014 and 2016.

Patent applications were similarly analyzed by searching the databases of the World Intellectual Property Organization (WIPO) [18], Brazilian Patent and Trademark Office (INPI) [19], Questel Intellectual Property Business Intelligence (Orbit software) [20], and European Patent Office (EPO) [21]. Claim searches using the “Zika virus” keywords generated 400 documents, with most of them mentioning potential applications in the treatment of Zika infections. These documents were analyzed individually for removal of duplicated data and only positive hits (*n* = 27) containing Zika virus as one of the application claims were kept in our analysis (S3 Table). The analysis of publication records and patent applications suggest that the intense scientific activity in the Zika virus field is still focused on basic research, as concluded from static trends of patent applications (Fig 1). It is worth noting that this observation is likely impacted by the fact that patent applications are generally published 18 months after the earliest date of the application and are confidential to patent offices prior to that date.

**Fig 2:**
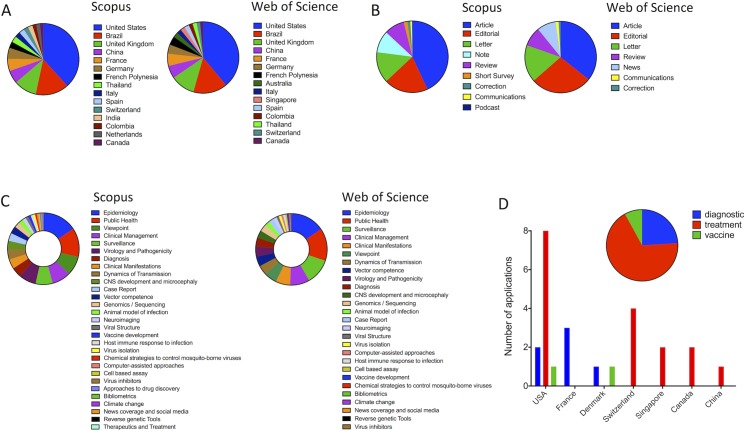
Classification of bibliometric indicators in the field of Zika virus. Scientific articles were classified according to author’s country affiliation (A), publication type (B), and research area (C). Both Scopus and Web of Science databases were used for this analysis. Article classification was performed manually using criteria that were established in previous studies [7]. Patent application (D) was classified according to the area of innovative activity and country where applications occurred. For analysis of raw data, see S1 and S2 Tables.

Scientific articles were mainly produced by authors affiliated with 18 countries (Fig 2A). Authors from the United States, Brazil, and the United Kingdom were the most frequent contributors. Most of these articles originated from regular research activity, but the health emergency also stimulated publication of letters and/or correspondences, editorials, news, and reviews (Fig 2B). Areas of research activity were apparently impacted by the obvious need of serological, therapeutic, and prophylactic tools, since Medicine and Immunology were by far the two principal topics of scientific activity, according to the Scopus categorization (Fig 2C). Patent applications, which produced numbers that were much more discrete than the records resulting from basic science (Fig 1), were distributed into drug discovery, diagnosis, and vaccine development, with the USA representing again the most active country (Fig 2D).

## Conclusions

On February 1, 2016, the World Health Organization declared the cluster of Zika-associated microcephaly cases and other neurological disorders a health emergency [22]. This action induced a Zika virus outbreak global response, and as of May 18th, 60 countries and territories were reporting continuing mosquito-borne transmission [23]. Additional international actions to combat the emergency were taken, as illustrated by the multiple international funding initiatives that are now available [24–27]. Although these international actions are all recent, new antiviral agents [28,29] and a vaccine platform protecting rhesus monkeys against the Zika virus challenge have been recently described [30]. This scenario is an example of the beneficial effects of continued generation of basic knowledge and innovation in the context of a health emergency.

## Supporting Information

S1 Table List of articles obtained from searches using the Scopus database. (XLSX)

S2 Table List of articles obtained from searches using the Web of Science database. (XLSX)

S3 Table List of patent claims containing the keyword “Zika.” (XLS)
